# Nitrogen cycling during secondary succession in Atlantic Forest of Bahia, Brazil

**DOI:** 10.1038/s41598-018-19403-0

**Published:** 2018-01-22

**Authors:** Joy B. Winbourne, Aida Feng, Lovinia Reynolds, Daniel Piotto, Meredith G. Hastings, Stephen Porder

**Affiliations:** 1 The Institute at Brown for Environment and Society, Brown University, Box 1951, Providence, RI 02912 USA; 2 Centro de Formação em Ciências Agroflorestais, Universidade Federal do Sul da Bahia, Ilhéus-BA, Brazil; 3 Department of Earth, Environmental, and Planetary Sciences, Brown University, Box 1846, Providence, RI 02912 USA

## Abstract

Carbon accumulation in tropical secondary forests may be limited in part by nitrogen (N) availability, but changes in N during tropical forest succession have rarely been quantified. We explored N cycle dynamics across a chronosequence of secondary tropical forests in the Mata Atlântica of Bahia, Brazil in order to understand how quickly the N cycle recuperates. We hypothesized that N fixation would decline over the course of succession as N availability and N gaseous losses increased. We measured N fixation, KCl-extractable N, net mineralization and nitrification, resin-strip sorbed N, gaseous N emissions and the soil δ^15^N in stands that were 20, 35, 50, and > 50 years old. Contrary to our initial hypothesis, we found no significant differences between stand ages in any measured variable. Our findings suggest that secondary forests in this region of the Atlantic forest reached pre-disturbance N cycling dynamics after just 20 years of succession. This result contrasts with previous study in the Amazon, where the N cycle recovered slowly after abandonment from pasture reaching pre-disturbance N cycling levels after ~50 years of succession. Our results suggest the pace of the N cycle, and perhaps tropical secondary forest, recovery, may vary regionally.

## Introduction

More than half of extant tropical forests are regenerating from disturbance^1^ and in the coming decades these re-growing forests will be a substantial carbon sink (~1 Pg yr^−1^)^2^, provide habitat for myriad species, and food, fuel and fiber for millions of people^2^. In the Neotropics, secondary forests recover biomass relatively quickly, reaching 90% of pre-disturbance biomass levels within ~66 years^3^. In part, the extent and pace at which secondary forests recover depends on the availability of essential plant nutrients, particularly nitrogen and phosphorus^4^. Land use activities driving deforestation, such as slash and burn agriculture, timber harvesting, and cattle grazing, remove essential plant nutrients such as nitrogen (N) and phosphorus (P) from the system^5,6^. While both N and P are lost during fires as particulates, N can also volatilize as nitrogen oxides (NO_x_) and nitrous oxide (N_2_O). This sets the stage for greater potential N than P limitation during early stages of secondary succession^6,7^. However, the possibility for high rates of biological N fixation suggests N may accumulate relatively quickly during secondary forest regrowth^8^. Both the absolute and relative availability of N and P in these systems is likely to influence the rate at which they recover^3–5,8^.

Biogeochemical theory along with empirical evidence suggests that mature lowland tropical forests on highly weathered soils tend to cycle N in relative excess compared with P^9,10^ with high N leaching^11^ and gaseous losses^12,13^. This is in part because phosphorus is ultimately sourced from parent material and gradually depleted over time^14^ in the absence of high dust inputs^15^ or rejuvenation by erosion^16^. In addition, the lowland neotropics have a relatively high (~10% basal area) abundance of trees and lianas in the *Fabaceae* family^17^ (hereafter “legumes”), many of which can form symbiotic relationships with N fixing bacteria. High legume abundance and relatively high levels of asymbiotic N fixation in the warm, wet litter and soil^18–21^, suggests inputs of N are 5–10 times higher than in temperate forests^21^. Thus relatively high N and low P availability may be common in the lowland neotropics, but this pattern can be altered when forests are cleared, since burning (a common land clearing strategy) preferentially removes N compared with P^6^.

To our knowledge, there has been one study that documented N recovery in secondary neotropical evergreen forests^7,22^ finding evidence for recuperation of the N cycle during ~70 years of secondary succession in the Brazilian Amazon. In this study, the response of several soil and plant metrics were examined (foliar δ^15^N, foliar N, litterfall N:P, soil nitrate, soil N_2_O emissions) across a chronosequence, finding that after ~50 years the N cycle transitioned from a relatively closed N cycle (low soil nitrate, low soil nitrate: ammonium ratio, low N_2_O losses) to a relatively open one (high soil nitrate, high soil nitrate: ammonium ratio, high N_2_O losses)^23^. Despite the apparent importance of N in limiting tree regrowth early in succession^22^, it is not known whether the pace of N recovery found in the Amazon is similar to that found elsewhere. There are several reasons to suspect that the N cycle trajectory during forest succession might differ even within neotropical forests. First, there is substantial variation in biomass recovery even in similar aged forest stands, both among and within secondary forest landscapes^3^. This suggests that different processes may regulate the pace of forest regeneration, and by extension regeneration of nutrient cycles. Part of this variation is attributable to climate^2^, but it is also likely that soils^24^, plant community assembly^25,26^, and past land use type and intensity^5^ are also important factors in N cycle recovery rates.

In this context, we studied N cycling across a chronosequence of secondary forests in the Brazilian Atlantic Forest (or the Mata Atlântica) in the south of Bahia, Brazil. Our study site is located in perhaps the least studied, and most threatened, tropical forest biome on earth^1^. This ancient forest, as diverse as the Amazon^27^, once stretched along the entire coastline of Brazil, but is now 85% deforested^1^. Some of the largest remaining fragments north of São Paulo state are found in the southern Bahia, and our study site is located in the Park Serra Do Conduru, which harbors the largest fragment of contiguous forest (10,000 ha of different aged forests) in the region. The Atlantic forest is a focus of extensive national and international restoration efforts^28^ and thus nutrient constraints to forest recovery have implications for both biogeochemists and restoration efforts. To our knowledge, our study represents the first comprehensive analysis of the N cycle in the Brazilian Atlantic Forest, and the second in the neotropics^7^.Our default hypothesis, in the absence of more data, is that the pattern observed in the Amazon would be the same in the Atlantic Forest. Thus we expect the N cycle would recover after ~ 50 years of forest succession. In order to test this hypothesis, we measured major inputs of N (via symbiotic and asymbiotic N fixation pathways), gaseous N losses (e.g. N_2_O & NO_x_), recycling (e.g. net mineralization and nitrification) and quantified plant available forms of soil N using two metrics.

## Results

### Nitrogen and phosphorus availability

**Figure 1:**
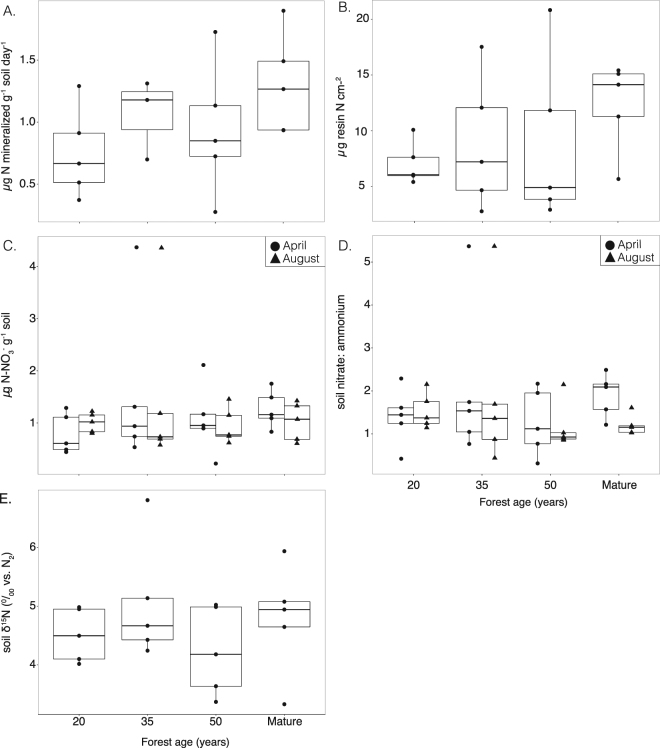
Indicators of nitrogen availability along secondary successional forest chronosequence. (**A**) Nitrogen mineralization rate, (**B**) *in-situ* resin extractable nitrate concentration, (**C**) instantaneous KCl-extractable soil nitrate concentration. and (**D**) soil nitrate: ammonium, (**E**) soil δ15N (^0^/_00_ vs. N_2_). Boxplot with each replicate plot indicated as a point (n = 5, except for mineralization rates in 35-year old forests, n = 3). Data is shown as boxplot with the median value indicated by the band near the middle of the box, the first and third quartiles are the top and bottom of the box, respectively, the maximum and minimum values excluding outliers are the whiskers. Data in panel C and D show data from two sampling times; April = closed circles and August = closed triangles. The effect of forest age is not significant for all N indicators examined (p > 0.05; K-W test).

**Figure 2:**
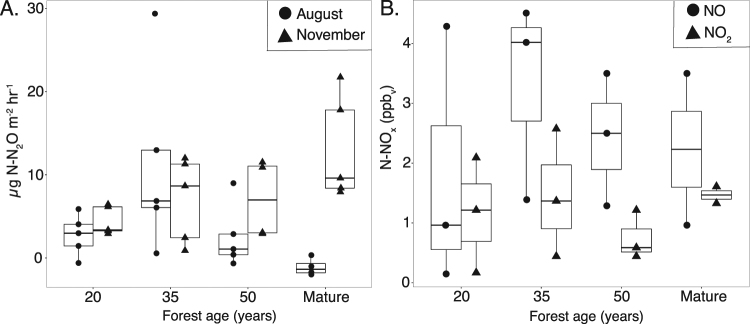
Nitrogen oxide soil emissions along secondary successional forest chronosequence. (**A**) nitrous oxide (N_2_O) emissions. Circles = August sampling and Triangles = November sampling (n = 5), and (**B**) nitrogen oxide emissions. Circles = NO and Triangles = NO_2_. (n = 3, except mature forest n = 2). Data is shown as boxplot with the median value indicated by the band near the middle of the box, the first and third quartiles are the top and bottom of the box, respectively, the maximum and minimum values excluding outliers are the whiskers. The effect of forest age is not significant for nitrogen oxide gases examined (p > 0.05; K-W test).

Inorganic N pools of ammonium and nitrate did not significantly vary with forest age in either April (p = 0.48, 0.61, respectively) or August (p = 0.67, 0.91, respectively; K-W test; Fig. 1C). The dominant form of inorganic N was nitrate with levels of ammonium frequently below detection (0.02 mg N- NH_4_^+^ kg^−1^). There was no difference between the concentration of nitrate (p = 0.88) and ammonium (p = 0.36) in August vs. April sampling (Fig. 1C,D). One site, from the 35-year-old forest, consistently had a higher concentration of nitrate near four times greater than all other sites. This site also had higher than average biomass for a 35-year-old forests according to biomass studies in these same study sites^29^, and ~3 times higher nitrous oxide flux than other 35-year-old forest sites (Fig. 2A). Analyzing data with this plot removed, however, did not change the overall lack of trend between inorganic N concentrations and forest age.

*In situ* measures of available nitrate, collected using the anion-exchange membranes, did not significantly vary with forest age (p = 0.44; K-W test; Fig. 1B). Resin strip phosphate levels were consistently close to, or below detection limit (0.05 mg P kg^−1^), with no significant difference among different-aged forests (p = 0.29; K-W test; Table 1).

**Table 1:** Site and soil characteristics. Soil data represents the top 10 cm of the mineral soil.

Forest Age	Soil carbon (g kg^−1^)	Soil nitrogen (g kg^−1^)	Resin extractable P (µg P cm^−3^ 2 weeks^−1^)	Soil pH	% legume basal area
20	33.3 (1.6) a	2.18 (0.099) a	0.0447 (0.0077) a	5.07 (0.031) a	37.3 (0.09) a
35	39.2 (2.7) a	2.66 (0.22) a	0.219 (0.13) a	4.84 (0.052) a	29.6 (0.09) a
50	37.9 (2.1) a	2.53 (0.16) a	0.361 (0.23) a	4.86 (0.11) a	33.2 (0.05) a
Mature	35.0 (2.7) a	2.28 (0.21) a	0.120 (0.093) a	5.15 (0.17) a	16.1 (0.03) b

Data is presented as the means with standard error in parentheses; n = 5. Letters indicate significant groupings (p < 0.05; Tukey or Nemenyi test; see methods for details).

Net N mineralization was dominated by net nitrification (>98%) and did not significantly vary with forest age (p = 0.20; K-W test; Fig. 1A). Total soil pools of N and carbon (Table 1) did not significantly change with stand age (p = 0.25 & 0.43, respectively; K-W test), nor were there significant differences in the N isotopic signature of soils with stand age (p = 0.58; K-W test; Fig. 1E). δ^15^N (0/_00_ vs. N_2_) of soil had a mean of 4.648 +/− 0.42 and ranged from 3.330 to 6.807.

### Nitrogen oxide gas fluxes

Nitrous oxide (N_2_O) fluxes were highly variable. In August, fluxes ranged from −1.95 µg N-N_2_O m^−2^ hr^−1^ to 29.36 µg N-N_2_O m^−2^ hr^−1^ (Fig. 2A). Mean flux in August was 4.09+/− 1.6 kg µg N-N_2_O m^−2^ hr^−1^. In November, fluxes ranged from 0 to 21.72 µg N-N_2_O m^−2^ hr^−1^. Mean flux in November was 7.95 +/− 1.3 µg N-N_2_O m^−2^ hr^−1^. Soil moisture, a key variable known to influence N_2_O fluxes^30^, was greater during the November sampling (p = 0.001). However, soil moisture did not explain significant variation in N_2_O fluxes in August (R = 0.1; p = 0.27) or November (R = −0.22; p = 0.89).

N_2_O fluxes did not significantly differ with forest age in either August or November (Fig. 2A). In August, the 35-year-old forests had significantly higher N_2_O fluxes than the mature forests (p = 0.01), but in November, there were no significant differences in N_2_O fluxes among forest age classes (p = 0.19). When mature forests were excluded, there were no significant differences observed in N_2_O fluxes among different aged secondary forests in either August (p = 0.14) or November (p = 0.09). There was no seasonal variation in N_2_O fluxes in 20-year-old (p = 0.3), 35-year-old (p = 0.80), and 50-year-old forests (p = 0.2; Wilcoxon test). However, N_2_O fluxes in mature forests differed between August and November (p = 0.007; Wilcoxon test) with a higher N_2_O flux in November.

Soil emissions of nitrogen oxide gases did not significantly vary between forests of different ages for either NO (p = 0.397) or NO_2_ (p = 0.498; Fig. 2B). N-NO fluxes ranged from 1.80 +/− 0.85 ppb_v_ in mature forests to 3.60 +/− 0.89 ppb_v_ in 35-year-old forests. N-NO_2_ fluxes were lower than NO fluxes and ranged from 0.748 +/− 0.24 ng ppb_v_ in 50-year-old forests to 1.421 +/−0.343 ppb_v_ in mature forests.

### Biological nitrogen fixation

**Figure 3:**
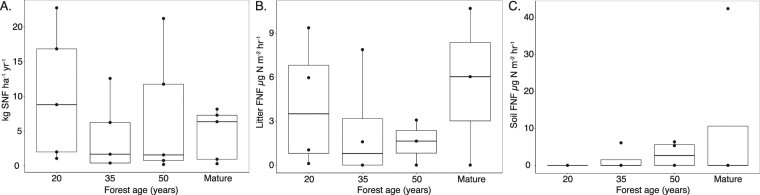
Biological nitrogen fixation along secondary successional forest chronosequence. (**A**) symbiotic nitrogen fixation (SNF), (**B**) leaf litter asymbiotic N fixation (FNF), and (**C**) soil asymbiotic N fixation. Data is shown as boxplot with the median value indicated by the band near the middle of the box, the first and third quartiles are the top and bottom of the box, respectively, the maximum and minimum values excluding outliers are the whiskers. The effect of forest age is not significant for symbiotic nitrogen fixation (p > 0.05; ANOVA) or asymbiotic nitrogen fixation p > 0.05; K-W test).

Despite differences in the abundance of legumes, which were significantly more abundant in the youngest forests (p < 0.05; Table 1), we found no significant differences in symbiotic N fixation across forest succession (F_(3, 16)_ = 0.51; p = 0.68; Fig. 3A). However, rates of SNF were most variable early in forest succession, ranging from 1.06 to 22.75 kg N ha^−1^ yr^−1^ in 20-year-old forests (mean 10.29 +/− 4.20 kg N ha^−1^ yr^−1^) compared to 0.3 to 8.16 kg N ha^−1^ yr^−1^ in mature forests (mean 4.61 +/− 1.7 kg N ha^−1^ yr^−1^). Additionally, there was no correlation between percentage of putative legume basal area and rates of SNF (R = 0.20; p = 0.16). Rates of asymbiotic N fixation did not significantly vary with forest age in either the soil layer (p = 0.53) or the leaf litter layer (p = 0.70; Fig. 3B,C). The fraction of total BNF accounted for by FNF varied across sites from 0–100% with no significant trends with stand age.

## Discussion

Contrary to our hypothesis we did not find evidence for changes in the N cycle during the 20 to 50 years of forest succession examined in this study. Instead our data suggests that secondary forests in this region of the Atlantic forest reach pre-disturbance N cycling dynamics by 20 years of succession. We measured several indicators of the N cycle including several different metrics of N availability (Fig. 1), N outputs via nitrogen oxide emissions (N_2_O, NO, and NO_2_; Fig. 2), and N inputs via symbiotic and asymbiotic N fixation (Fig. 3) - none of which varied significantly with forest age in our chronosequence, nor between all secondary forests versus mature forests. These findings are in contrast to a similar study^7^ in the Brazilian Amazon where N cycle indicators increased over the course of 3 to 70 years of succession, a time frame that includes the period examined in this study (20 to 50 years). Given the abundance of legumes and the high soil nitrate: ammonium, we hypothesize that the N cycle recovered rapidly in the first 20 years of succession. Future studies in this region, however, are needed in young forests (<20 years) to test this hypothesis.

In the absence of other major external N sources (such as anthropogenic nitrogen deposition^31^), biological N fixation especially by legume-rhizobia symbiosis, is thought to be the main pathway for secondary forests to recuperate N losses from prior land use activities and meet the N demands of rapidly re-growing forests^8^. Rates of SNF in excess of ~10 kg N ha^−1^ yr^−1^ could account for the accumulation of N in biomass observed during succession^8,21^. Available methods for determining rates of biological N fixation are, however, poorly constrained and have their limitations^32^. Nevertheless, the few studies to quantify N fixation by legume species during secondary succession suggest SNF rates are highest in the first twenty years of forest succession^8,21^, resulting in increased pools of inorganic N, N mineralization rates, and N_2_O fluxes^33–35^. While we did not observe a significant difference in SNF with stand age we did observe the highest rates in some of the youngest forests (upwards of 20 kg N ha^−1^ yr^−1^), and it is possible that with greater sampling effort we would have more support for the trend towards higher SNF in younger sites. Alternatively, if even ~10% of the recalcitrant forms of soil N were mineralized gradually over time (in top 10 cm of mineral soil there is ~1872 kg N ha^−1^), then ample N would be available to fuel the biomass recovery observed in study plots^29^.

Three lines of evidence suggests the N cycle might recover quickly in the first 20 years of succession (Fig. 1). First the ratio of soil nitrate to ammonium is high suggesting soil N has accumulated in excess of plant demand^36^. This is because ammonium is often the dominant form of inorganic soil N in N-limited systems^7^. Ammonium is the precursor to the production of nitrate during the mineralization of organic matter. Therefore, increases in the soil nitrate pool tend to only occur once ammonium has accumulated in the soil allowing nitrifying bacteria to become competitive and convert ammonium into nitrate^36^. Indeed, nitrification was found to be the dominant (>98%) process responsible for mineralization across forest stand ages. Together these two pieces of evidence suggest that N supplies are sufficient for biological demand, even in 20-year old forest stands. Lastly, the lack of a significant change in nitrogen oxide emissions (N_2_O, NO, NO_2_) with forest age is also consistent with this hypothesis. The aerobic process of nitrification and anaerobic process of denitrification are the main contributors to nitrogen gas losses, both of which require inorganic N pools in the soil to accumulate past biotic demand (or for microbes to outcompete plants for inorganic N)^37^. Increases in nitrogen oxide gases thus indicate the recuperation of several N cycle processes. However, nitrous oxide fluxes are known to be highly variable and therefore the lack of a trend with forest age could also be due to insufficient temporal and spatial sampling^38^. Nevertheless, together these data suggest N has re-accumulated in this system after just 20 years of forest succession. Future studies, however, are needed in the region on forests <20 years to test this hypothesis. Unfortunately, in our study area, owing to the age of the park, forests <20 years old were not available.

Given differences in methods for quantifying N cycle metrics (e.g. different extraction times and degree of shacking in KCl), we were only able to compare our study system to forests on the Osa Peninsula of Costa Rica^39^. We found our study system had levels of KCl-extractable N, nitrification rates and mineralization rates on the low end of those relatively N poor mature forests in the Osa Pennisula^39–41^. However, the ratio of nitrate:ammonium in our dataset suggests N is accumulating and meeting plant demands. The naturally low N levels observed in this region of the Brazilian Atlantic forests may explain a potential for rapid recovery of the N cycle to pre-disturbance levels. While mature lowland tropical forests are thought to cycle N in excess relative to rock-derived nutrients such as P, recent studies have demonstrated that tropical lowland forests have a wide range of N availability^39^ at multiple spatial scales^42^. Overall, our study provides support to a growing body of evidence demonstrating the heterogeneity in tropical forest N cycling at multiple spatial scales^42,43^.

Differences in prior land use type and intensity, as well as climatic differences in water availability, fire regimes and soil types, may explain the N cycle trajectories observed in this study compared to prior work in the Brazilian Amazon. In our study, we focused on secondary forests growing on areas that experienced slash and burn agriculture with short cultivation phase (2–3 years) while previous work in the Amazon focused on land re-growing on both abandoned pastures and cropland. These two types of land use practices are common in tropical regions and are known to change the initial starting conditions of soil nutrients^5^; however, pasture management leads to increased soil compaction than non-animal mechanized agriculture^44^. Intensive prior land use by either practice can inhibit the establishment and growth of woody vegetation during early stages of succession, such as legume tree species, thereby delaying the recovery of the N cycle^5^. Indeed, legumes are not observed as the dominant species in some young successional forests of the eastern Amazon^22^. A recent meta-analysis of tropical secondary forest biomass recovery, however, found that there were no significant differences in the biomass recovery between slash and burn agriculture versus pasture^3^. The authors suggest this may be a result of the high within-category variation observed in land use intensity. Instead differences in biomass recovery were more strongly influenced by variation in water availability, both higher annual rainfall and lower climatic water deficit. In Bahia, rainfall is aseasonal and natural fires are uncommon^45^. In contrast, the study in the Amazon was in a region that experienced similar rainfall (~1800 mm/year) but with a strong dry season and history of natural forest fires^7,22^.

## Conclusion

Our study examined N cycle dynamics across a chronosequence of secondary tropical forests in the Mata Atlântica of Bahia, Brazil in order to understand how quickly the N cycle recuperates. The pace at which the N cycle recovers has important implications for restoration efforts and conservation priorities. We found that the Atlantic forest reached pre-disturbance N cycling dynamics after just 20 years of succession. These findings suggest that the N cycle recuperates quickly in the first 20 years of succession, however, additional studies in younger forests within the region (<20-year-old stands) are needed. To our knowledge, our study represents the first comprehensive analysis of the N cycle in the Brazilian Atlantic Forest, and the second in the neotropics^7^. Our study supports emerging evidence that symbiotic nitrogen fixation helps the N cycle recover during secondary succession^8,21^. However, the pace at which the N cycle recovers appears to vary across tropical secondary forests highlighting the need for future studies to test the controls and factors that influence the pace of N cycle recovery during tropical secondary forests succession.

## Methods

### Study Site

Our study sites are secondary and mature forests in and around Serra do Condoru State park located in southern Bahia, Brazil (14°25′ S and 39°05′ W) within established forest plots^45,46^. The park was established in 1997 and encompasses an area of roughly 10,000 ha, the largest contiguous area of Atlantic Forest north of São Paulo state. Forests in the park are a mosaic of different stages of development including secondary forests and remnants of mature forests that have varying degrees of selective logging in the past. During our study period (April to November 2016) mean annual temperature at the site was 24 °C and mean annual precipitation was ~200 mm of rainfall per month. The area does not have significant seasonality in temperature or rainfall except during strong El Niño years when monthly rainfall was typically <25 mm. Our sampling begun at the end of an El Niño year.

Chronosequence – We sampled in 20, 10 × 50-meter study plots consisting of four age classes 20, 35, 50, and mature, with 5 replicate plots per age class. Sites were selected to control for previous land use (slash and burn agriculture followed by 1 to 2 years of manioc cultivation) and differences in soil types (Typic Haplorthox), while including regional variation in topography (120–300 m above sea level and 10–30% slope angle). The estimation of site ages and past land uses were based on a sequence of available aerial photographs and remote sensing data which provided precise and verifiable estimates of site age. Changes in forest cover and land use in a 5000-ha area inside Serra do Conduru State Park were estimated from 1965 to 2007. The remote sensing windows were based on aerial photos taken in 1965, 1975, 1986, 1997, 2002, and 2007. Land use maps were generated for every set of aerial photos and maps of forest age classes were derived using GIS. Information on the type and intensity of past land use was gathered by interviewing local farmers. A total of 95 secondary forest stands (ages ranging from 20 to 50 years old) larger than 3-ha and adjacent to a mature forest were located in the study area. Study plots were randomly selected to represent 5 replicates of four age classes: 20, 35, 50 and >50 years old (or mature). Because the Serra do Conduru state park was legally created in 1997, there were no portions of the park on recently abandoned agricultural fields, which constrained the inclusion of stands younger than 20 years in the chronosequence. Mature forests in the region varied in the degree of human disturbance (e.g. selective logging and hunting) and we selected mature forests sites that captured this variation, as indicated by the range in aboveground biomass for mature forests (300–500 Mg ha^−1^). At each site, secondary forest plots were located 20 to 50 m from a mature forest fragment and mature forests were at least 10 m from a secondary forest edge. A census for tree size and species identification was done for all plots in 2009. For more information on establishment of study plots see Piotto *et al*.^45,46^.

### Soil nitrogen and phosphorus availability

We quantified N availability using three techniques: 1) instantaneous 2 N potassium chloride (KCl) extractable inorganic N (NH_4_^+^, NO_3_^−^), 2) net N mineralization and nitrification, and 3) cumulative *in situ* extractable NO_3_^−^ measured using anion exchange resins over a period of two weeks. We measured instantaneous extractable inorganic N in April and August of 2016, while all other measurements were collected only in August 2016. For the analysis of inorganic N pools, net mineralization and net nitrification rates, we collected soils (3 per plot) from the top 10 cm of mineral soil using square 100 cm^2^ core. Within 4 hours of collection we sieved (2 mm) each soil core, extracted 8-g of soil in 30 mL of 2 N KCl and filtered through Whatman No. 1 filter paper. Net N mineralization and net nitrification were measured from changes in NH_4_^+^ and NO_3_^−^ concentrations during a 4-day aerobic incubation of ~8-g subsamples in the dark at field temperatures. Each extraction was shaken manually for 60 seconds every hour for 4 hours^39,47^. All extracts were stored frozen until analyzed. ~10 g subsample was dried at 65 °C for 3 days to determine soil moisture. All measurements are reported on a dry-mass basis.

We used anion exchange resins membranes to determine mobile nitrate and phosphate *in situ* over the course of a two-week period. We prepared anion-exchange membrane strips using sheets of cross-linked copolymers of vinyl monomers from (General Electric #AR204SZRA). We prepared strips (~2 × 10 cm) by shaking them for 24 hours in 0.5 M NaHCO_3_ for phosphate, and 1 M NaCl for nitrate^48^. We inserted 12 anion-exchange membranes (6 nitrate, 6 phosphate) into the top 10 cm of each plot. The strips were inserted at a ~45° angle relative to the landscape slope and left in the field for 14 days. After 14 days in the field, we collected the membranes and rinsed them gently with distilled water to remove residual soil. The strips were kept at 4 °C and transported to the laboratory at Brown University. We shook the resin strips for 4 hours in either 30 mL of 2 M KCl solution (for nitrate) or 30 mL of 0.5 M HCl (for phosphate). We then filtered the samples through Swinnex filters and frozen until analysis. The area of the resin strip was determined using ImageJ software, and we report results as nutrient concentration per centimeter squared of resin strips. Concentrations of ammonium, nitrate, and phosphate from extracted samples were analyzed colorimetrically on a Westco SmartChem 200 Discrete Element Analyzer (Westco Instruments, Brookfield, Connecticut).

### Soil Properties

In April 2016, three replicate cores per plot (1000 cm^3^) were collected, passed through a 2-mm sieve, and dried at 65 °C. Soils were sent to the Stable Isotope Facility at the University of California, Davis for analysis of total carbon and nitrogen, as well as the nitrogen isotopic signature. Soil pH and bulk density were quantified when the plots were established in 2009^45,46^.

### Soil nitrogen oxide gas emissions

We measured soil emissions of N_2_O fluxes in August and November of 2016 using syringe sampling of vented static chambers^49^ made of septic PVC (20 cm diameter) and placed in the soil to a depth of 2 cm after removing leaf litter from the surface. Three replicate chambers were placed in each of the 20 study plots. We waited 25 minutes after installing the collars before the first headspace sample was collected thus allowing for soil emissions to equilibrate after the disturbance created by inserting the collar. We then collected headspace samples every 15 to 45 minutes (a total of 4 time points). To reduce the effect of diel variability, we collected gas samples between the hours of 9:00 and 14:00 hour local time and varied the time at which different aged plots were sampled (for example, within an age class at least one replicate plot was sampled in the morning, mid-day, and afternoon).

All N_2_O gas samples (20 ml) were stored over pressurized in 12 mL Labco exetainer vials and returned to the lab at Brown University for analysis on Shimadzu GC-2014 equipped with electron capture detector (ECD) for quantifying N_2_O and flame ionization detector (FID) for quantifying CO_2_. We used the accumulation of CO_2_ during the 45 minutes the collar was capped to check for leaks in the collars. In addition, we used a set of standards to test for sample leakage, which traveled with the samples.

For both N_2_O and CO_2_ we calculated fluxes from linear regressions of the concentration increase, chamber volume, and air temperature and pressure (collected from the nearby Ilhéus Jorge Amado Airport weather station^50^) at time of sampling. The low N_2_O fluxes required longer deployment time (45 minutes) to reach linear increases in [N_2_O] over time. We used the degree to which [CO_2_] over time was linear as an indicator of potential chambers leaking. We therefore, did not include samples in our analysis if the [CO_2_] did not increase over time with a linear R^2^ > 0.98. However, if [CO_2_] increased linearly over 30 minutes these samples were retained in the analysis, with emissions rates based on 30 minutes. In total 30 out of 120 replicates were removed, and 10 additional 45-minute time points. N_2_O fluxes are presented as µg N-N_2_O m-^2^ hr^−1^ following equations in ref.^49^.

Soil moisture (an important covariate to consider with N_2_O fluxes^30^) was determined at the time of sampling using gravimetric methods in August 2016, and a TDR soil moisture probe (FieldScout TDR 300 #6430FS, Spectrum Technologies Inc.) in November 2016. N_2_O fluxes were coupled with measures of inorganic N for August 2016 sampling dates but not for November 2016. Inorganic N pools were not found to vary seasonally in our plots between April and August sampling dates, and logistical challenges prevented inorganic pools from being sampled in November 2016.

We quantified soil emissions of NO_x_ using ambient air passive samplers consisting of pads that chemically captured NO_2_ and NO_x_ (NO and NO_2_) (Ogawa & Co.). Samplers were deployed using a PVC rain shelter and support clips provided by the manufacturer and placed ~ 0.3 meters above the soil surface attached to a small diameter (<5 cm) tree. A set of blanks (pads stored in gas tight brown Nalgene bottles) were carried along with samples to determine potential NO_x_ collection during transport and storage. Samplers were collected after 10 weeks of exposure, and pads were extracted in 8 ml of double distilled water which converted captured nitrogen oxide gases into nitrite. The resulting solution was kept refrigerated prior to analysis for the concentration of nitrogen as nitrite on a Westco SmartChem 200 Discrete Element Analyzer (Westco Instruments, Brookfield, Connecticut). Prior to chemical analysis, we treated the extraction of NO_x_ pads with 1:1 ratio of sample to ether in order to remove a blue dye contained in the pads that interferes with colorimetric analysis of ion concentrations^51^. The ether precipitates the dye into a supernatant layer that was carefully removed with a pipette and the remaining ether was allowed to evaporate^51^. Following the protocol by Ogawa & Co., ambient gas phase N-NO_2_ in ppbv were calculated as C = (Q_NO2_ * $\propto$_NO2_)/t, where C = concentration in ppbv, Q_NO2_ = N-NO_2_ mass in ng, $\propto$_NO2_ = sampling rate, and t = sampling time in minutes. Sampling rate ($\propto )$ is dependent on ambient pressure, temperature and humidity, and are determined based upon lookup tables provided by Ogawa. We used the average relative humidity (80%) and average temperature (25 °C) during the 10 weeks of exposure to determine the sampling rate. Similarly, the ambient N-NO concentration was calculated as C = ((Q_NO2_ − Q_NO2_)) * $\propto$_NO_)/t, where Q_NOx_ is the mass of N-NO_x_ in ng. Data is presented as N-NO or N-NO_2_ in units of ppb_v._ NO estimates are not sensitive to changes in temperature, however, a 10% change in relative humidity results in 4% change in NO concentrations. NO_2_ estimates are sensitive to both temperature and relative humidity changes; a 5 °C difference in temperature results in 5.5% change in concentrations, while a 10% change in relative humidity results in 3% change in concentrations.

### Symbiotic and asymbiotic N fixation

We measured symbiotic rates of N fixation randomly across 20 study plots over the course of 4 weeks in April and early May of 2016. Quantifying SNF requires destructive harvesting and is time consuming thus preventing seasonal measurements. We measured asymbiotic fixation in all 20 plots over the course of one week in April of 2016. Rates of N fixation were coupled with measures of inorganic N pools but were not also coupled with nitrous oxide gas emission sampling efforts due to logistical constraints. We quantified N fixation rates using traditional acetylene reduction assay (ARA) which were calibrated with ^15^N_2_ gas incubations for symbiotic nitrogen fixation (details below). We measured rates of symbiotic N fixation using a modified stratified adaptive cluster sampling approach (SACS) described by (Sullivan *et al*.^21^) to estimate both nodule biomass and rates of N fixation. Briefly, in each plot we sampled every 2 meters along 5, 10 m transects for a total of 25 cores per plot. When nodules were present in a core, all adjacent cores were examined for nodules until no more nodules were found. We used a 100 cm^2^ soil core to a depth of 10 cm. All excised nodules found using the SACS method were immediately placed in a 60-ml gas tight jar fitted with septa, exposed to 10% atmosphere of acetylene, and incubated for 1 hour. Acetylene was made in the field using calcium carbide. With each set of ARA, a set of blanks (acetylene with no sample) and controls (sample, no acetylene) were established to determine background ethylene levels present in the acetylene or produced by the sample. Within hours of field collection, nodules were desiccated using silica gel before weighing dry mass.

We measured asymbiotic fixation on decomposing leaf litter and the top 2 cm of the mineral soil horizon. We collected decomposing leaf litter from across the plot area (10 m × 50 m) and homogenized by hand before weighing roughly 2.5 grams of fresh litter into 60 ml acrylic tubes fitted with septa (6 replicates per plot). Soil was collected using a soil core that was 2 cm in diameter. As with excised nodules, soils and litter were exposed to 10% atmosphere of acetylene. Asymbiotic N fixation samples were incubated at field conditions for 18 hours because of the low rates of asymbiotic N fixation. The dry mass and moisture content of soil and litter samples were determined gravimetrically after oven drying for 3 days at 65 °C.

All gas samples from ARA method were stored in pre-evacuated gas tight jars (Labco, Exetainers), and returned to Brown University for analysis of ethylene concentrations using a Shimadzu GC-2014 gas chromatograph equipped with a flame ionization detector (330 °C) and a Poropak-T column at 80 °C. Rates of ARA were calculated as nanomoles ethylene produced dry g^−1^ hour^−1^. Rates of acetylene reduced by symbiotic N fixers were converted to N_2_ fixed using the site specific conversion ratio (2.98 mol ethylene reduced:1 mol N_2_ fixed) determined by ^15^N_2_ incubations following methods described in ref.^52^. Rates of SNF are reported as kg N fixed ha^−1^ yr^−^^1^ assuming 24 hours of BNF activity year round, thus allowing for comparisons in the literature. Asymbiotic rates were converted to N_2_ fixed using the theoretical conversion ratio of 3:1. Measures of asymbiotic N fixation were scaled to an area-based flux (e.g. µg N m^−1^ hr^−1^) by measuring the mass of litter on the forest floor in a defined area, at the time of collection.

### Statistical analyses

All statistical tests were performed on the average of technical replicates from each plot. Data were tested for normality using Shapiro-Wilk test and homoscedasticity using Levene’s test. A 1-way ANOVA on ln-transformed data was used to determine the effect of forest age on rates of symbiotic N fixation, soil C%, soil N%, pH, and legume abundance. All other metrics of the N cycle violated the assumptions of parametric tests even after being ln-transformed, and thus non-parametric Kruskal-Wallis test on the untransformed data was used to determine significant effect of forest age on all N cycle metrics (e.g. inorganic N pools, mineralization rates, resin available P, resin available N, nitrogen oxide emissions, asymbiotic N fixation). A post-hoc Tukey test or Nemenyi test was used to determine significant groupings for data analyzed using parametric versus non-parametric statistics, respectively. Seasonal variation in inorganic N pools and nitrous oxide fluxes were determined using non-parametric t-test, Wilcoxon rank-sum. Statistical analyses were performed using R software v.3.0.2 (R Development Core Team).
